# Museum, Atlanta Medical Men, &c

**Published:** 1856-05

**Authors:** 


### MUSEUM, ATLANTA MEDICAL MEN, &C.

The Atlanta Medical College is just in receipt of a very handsome present for the Museum, from Dr. N. D'Alvigny, which he has collected with much care during the fifteen years he has spent in the practice of regular and dental surgery, in the United States. The collection consists, in part, of some beautiful specimens of morbid anatomy of the bones, a variety of specimens of surgical accidents and diseases generally, and the most perfect specimens of morbid dental anatomy we have ever seen, are among the collection.

Beautiful casts, in white wax, of various deformities, taken before and after an operation; showing the precise appearance before the attempt to restore the part to its natural condition, and the improvement effected by it, together with a description of the mode of operation. Some very interesting monstrosities, both human and animal, with several articles belonging to the study of natural science. Would that the taste of more members of our profession led them to preserve such collections, with their history, including treatment, symptoms, &c.

Dr. J. Gilbert and Dr. T. C. H. Wilson also presented some rare specimens of monstrosity, and other obstetrical preparations. In point of curiosity, some of these are rarely surpassed.

To all of these gentlemen the thanks of those connected with the Institution are rendered; and the unsolicited donation of such valuable material for the museum shows a praiseworthy disposition to encourage the effort we are making, to present the fullest advantages for an acquisition of a thorough knowledge of medical science. J. G. W.

We would remark upon the above, that those who are engaged in the college enterprise appreciate the generous countenance and aid which is being manifested by the regular profession of the city, not *one* of whom, so far as we know, having any other than the most friendly interest in its success.

And we would take this occasion to say, that after some opportunity of observation in reference to medical intercoursé elsewhere, we have yet to find the point, where *so much* good feeling prevails among the profession, as in the city of Atlanta —a fact, than which, few could be stated more creditable to the heads and hearts of our medical men.

In addition to this, we are happy to know that there is a spirit of investigation and a desire for the advancement of science which is not to be found in many an older city, and which is rapidly placing the medical men here upon an equal footing with those to be found in any part of the land.

And why may it not be, that Atlanta should be able to furnish, not only her own citizens with medical advice and relief, equal to that to be obtained any where, but that the reputation and skill of her physicians and surgeons should be able to arrest the annual tide of invalids from the South, who are wending their way, for relief, to an inhospitable clime, and still more inhospitable hearts ?—*Editors.*

